# Effectiveness of a Web-Based Cognitive-Behavioral Tool to Improve Mental Well-Being in the General Population: Randomized Controlled Trial

**DOI:** 10.2196/jmir.2240

**Published:** 2013-01-09

**Authors:** John Powell, Thomas Hamborg, Nigel Stallard, Amanda Burls, Jaime McSorley, Kylie Bennett, Kathleen M Griffiths, Helen Christensen

**Affiliations:** ^1^Department of Primary Care Health SciencesUniversity of OxfordOxfordUnited Kingdom; ^2^Division of Health SciencesWarwick Medical SchoolUniversity of WarwickCoventryUnited Kingdom; ^3^NHS ChoicesDepartment of HealthLondonUnited Kingdom; ^4^Centre for Mental Health ResearchThe Australian National UniversityCanberraAustralia; ^5^Black Dog InstituteUniversity of New South WalesRandwickAustralia

**Keywords:** Mental health, Public health, Randomized controlled trial, Internet

## Abstract

**Background:**

Interventions to promote mental well-being can bring benefits to the individual and to society. The Internet can facilitate the large-scale and low-cost delivery of individually targeted health promoting interventions.

**Objective:**

To evaluate the effectiveness of a self-directed Internet-delivered cognitive-behavioral skills training tool in improving mental well-being in a population sample.

**Methods:**

This was a randomized trial with a waiting-list control. Using advertisements on a national health portal and through its mailing list, we recruited 3070 participants aged 18 or over, resident in England, and willing to give their email address and access a fully automated Web-based intervention. The intervention (MoodGYM) consisted of 5 interactive modules that teach cognitive-behavioral principles. Participants in the intervention arm received weekly email reminders to access the intervention. The control group received access to the intervention after the trial was completed and received no specific intervention or email reminders. Outcomes were assessed by using self-completion questionnaires. The primary outcome was mental well-being measured with the Warwick-Edinburgh Mental Well-being Scale (WEMWBS). Secondary outcomes were Center for Epidemiologic Studies Depression scale (CES-D) depression scores, Generalized Anxiety Disorder 7-item scale (GAD-7) anxiety scores, EuroQol Group 5-Dimension Self-Report Questionnaire (EQ-5D) quality of life scores, physical activity, and health service use. All outcomes were measured at baseline, and at 6- and 12-week follow-ups.

**Results:**

A total of 1529 (49.80%) participants completed final follow-up at 12 weeks. Retention was 73.11% (1123/1536) in the control arm and 26.47% (406/1534) in the intervention arm. No relationship between baseline measures and withdrawal could be established. The analysis of WEMWBS mental well-being scores using a linear mixed model for repeated measures showed no difference between intervention and control group at baseline (difference –0.124 points, 95% CI –0.814 to 0.566), and significant improvements for the intervention group at 6 weeks (2.542 points, 95% CI 1.693-3.390) and at 12 weeks (2.876 points, 95% CI 1.933-3.819). The model showed a highly significant (*P*<.001) intervention by time interaction effect. There were also significant improvements in self-rated scores of depression and anxiety. Given the high level of attrition, a sensitivity analysis with imputed missing values was undertaken that also showed a significant positive effect of the intervention.

**Conclusions:**

Participants allocated to the intervention arm had an average increase of approximately 3 points on the WEMWBS scale compared to no increase for participants in the control group. Three points on this scale is approximately one-third of a standard deviation. In a low-cost automated intervention designed to shift the population distribution of mental well-being, a small difference per individual could yield a major benefit in population terms. In common with other Web-based interventions, there were high rates of attrition. Further work is needed to improve acceptability, to evaluate against placebo effect, and to disaggregate the effect on mental well-being from the effect on depression and anxiety.

**Trial Registration:**

International Standard Randomised Controlled Trial Number Register ISRCTN 48134476; http://www.controlled-trials.com/ISRCTN48134476 (Archived by WebCite® at http://www.webcitation.org/6DFgW2p3Q)

## Introduction

Interventions to promote positive mental health and well-being can bring benefits both to the individual by improving mood and psychological functioning, and also to society in terms of economic prosperity and social cohesion [1]. There is now worldwide interest in the promotion of mental well-being with measures of well-being being adopted as key economic indicators alongside gross domestic product (GDP) [2]. Yet there are few studies of individually targeted interventions with a primary aim of promoting mental well-being. In theory, an approach using the principles of cognitive behavioral therapy (CBT) to encourage more healthy patterns of thinking and behavior may offer an individual-level intervention to promote positive mental health. There is evidence for the effectiveness of CBT approaches in preventing depression (primarily among adolescents and young adults) [3-5], in improving resilience (often group interventions delivered in workplace settings) [6], and in the promotion of workplace well-being yielding benefits such as improvements in productivity, sickness absence, and stress [7]. There is also an emergent literature on the promotion of well-being using positive psychology interventions that encompass a range of psychological approaches including cognitive-behavioral aspects [8].

At the same time, the Internet is playing an increasingly important role in health care. It can provide a platform for the large-scale delivery of information and interventions for modifying lifestyle risk factors that result in more informed and empowered citizens who are better able to manage their own health. The area of e-mental health has been of particular interest to researchers and practitioners [9] because online tools, such as Internet-delivered computerized cognitive behavioral therapies (CCBT), have been shown to be effective for a range of mental health conditions [10], both when combined with therapist contact and when fully automated [11]. The Internet is also being used for positive psychology approaches [12]. The fully automated Web-based MoodGYM intervention was originally developed as a tool to prevent depression in young people and has been demonstrated to be effective in this context [13]. It has also been shown to be acceptable, safe, effective, and cost-effective in alleviating symptoms of mild to moderate depression and anxiety in community samples [14-17]. Although self-directed Internet interventions are known to have low rates of adherence [18], this is less of a problem in well-being promotion for the general population than for the treatment of mental illness because it does not raise ethical questions of inadequacy of treatment for a diagnosed health problem. Moreover, as a mental health promotion tool, the intervention can be delivered at very low marginal cost by using minimal personnel resources so that it can be made freely available to all who wish to use it, in contrast to a therapist contact approach that is neither feasible nor affordable for all.

In this study, we undertook a randomized controlled trial to test the effectiveness of a Web-based individually targeted self-help CBT package (MoodGYM) for promoting mental well-being in the general population.

## Methods

### Study Design and Participants

We undertook a randomized trial with two parallel group arms: intervention and a waiting-list control. Recruitment took place over 2 weeks in September 2010. Participants were self-recruited users of the UK National Health Service (NHS) NHS Choices website who were invited to take part in an online trial to promote mental well-being. Self-completion pop-up user surveys conducted previously showed that in 2010 most users of this NHS Choices website were women (76%), and 68% of users were in the 25 to 64 years age range. To be eligible for our study, participants were required to confirm that they were aged 18 or over, lived in England (as covered by our ethics and governance approval), and had Internet access and an email address.

### Procedures

Study recruitment advertisements were placed on the NHS Choices website (specifically the Live Well and mental health pages), in the NHS Choices newsletter sent to all subscribers (approximately 80,000), in emails sent to NHS Choices Customer Insights research group, and on the NHS Choices Facebook and Twitter pages, as well as on the Carers Direct Facebook page. These advertisements offered participants the opportunity to take part in a mental fitness trial, with the aim of promoting mental well-being. The study was not advertised as a treatment for people who were ill; the emphasis was on mental health promotion. Those interested in participating were invited to complete an online form to confirm eligibility, and to read information about the study. After a period of 48 hours to allow them time to reflect on their decision to take part in the research, eligible participants were invited by email to provide informed consent via an online form, to create a username and password, and to complete baseline questionnaires. Trial administration was automated and participants remained quasi-anonymous, identified only by email address. Multiple registrations by single email address were forbidden.

After completion of baseline measures, participants were sent an automated email directing them to log in to a trial portal with their new username and password. At this point participants were automatically randomized to either the intervention or control group. Once randomized, participants were immediately provided with access to the intervention (intervention group) or they were given general information about accessing the NHS Choices Healthy Living pages and informed that they would receive the intervention after a period of 3 months (waiting-list control group). Randomization was in a 1:1 ratio using predefined automated computerized block randomization with a block size of 2. The automated computerized system was set up by technical staff not involved in the day-to-day management of the study. Allocation was concealed from the researchers. As we chose to use a waiting-list control, participants were not blind to whether or not they were in the intervention group. To prevent contamination in the control arm, we did not use the name “MoodGYM” in the study documentation. Participants were free to withdraw at any time without giving a reason. There were no content changes, periods of downtime, or bug fixes required during the trial.

We received approvals from the NHS ethics committee (Black Country REC 10/H1202/21), the Australian National University (ANU) Human Research Ethics Committee (protocol number 2010/244) and NHS research governance. The study was registered on the International Standard Randomised Controlled Trial Number Register (ISRCTN 48134476).

### Intervention

**Figure 1:**
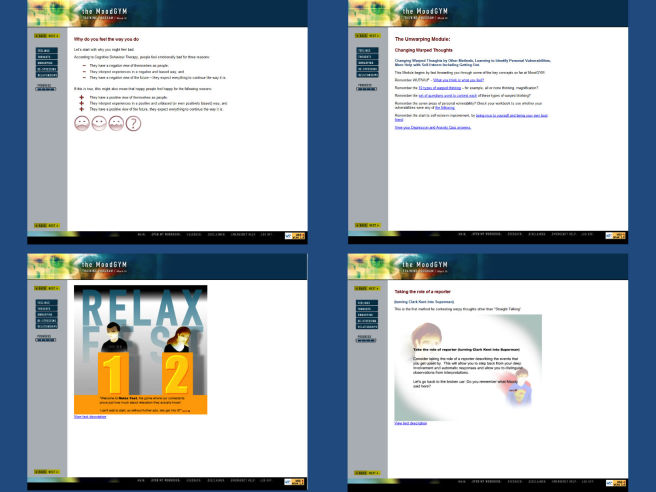
Screenshots of MoodGYM intervention.

MoodGYM is a free Internet-based self-help program that teaches cognitive-behavioral skills. It consists of 5 interactive modules that use diagrams and online exercises. It demonstrates the relationship between thoughts and emotions, examines issues related to stress and to relationships, and teaches relaxation and meditation techniques. It also includes sections on managing relationships and problem solving. Screenshots of the MoodGYM intervention are shown in Figure 1. Participants are encouraged to work their way through each of the 5 modules, 1 module per week, but are able to work at their own pace, ad libitum. The program includes an online workbook with 29 online exercises to help promote mental health. We made slight modifications to some phrases used in the MoodGYM tool to replace Australian colloquialisms with their English equivalent. We added logos to indicate affiliation to the NHS and University of Warwick (lead academic institution). Participants in the intervention arm received weekly email reminders to log in to the trial portal where they could access the intervention.

### Control Group

The comparator was a waiting-list control group. During the trial, the control participants did not receive any specific intervention or email reminders. In common with the participants in the intervention arm, control participants were able to access general information pages on mental well-being on the NHS Choices website. At the completion of the trial (3 months after its commencement), participants in the control group were provided with access to the intervention.

### Outcome Measures

The primary outcome measure was mental well-being as measured using the self-completion Warwick-Edinburgh Mental Well-being Scale (WEMWBS) [19]. This 14-item instrument has been validated for the UK population and adopted by the Scottish Health Survey and the Health Survey for England. It asks respondents to read statements about feelings and thoughts and to choose the response (a 5-point scale ranging from none of the time to all of the time) that best describes their experience over the previous two weeks. Example items are “I’ve been feeling optimistic about the future” and “I’ve been thinking clearly.” It has been shown to have good content validity and shows high correlations with other scales of mental health and well-being. It has a near-normal population distribution, with no ceiling effects.

Secondary outcomes were self-completed Center for Epidemiologic Studies Depression scale (CES-D) depression scores, Generalized Anxiety Disorder 7-item (GAD-7) anxiety scores, EuroQol Group 5-Dimension Self-Report Questionnaire (EQ-5D) quality of life scores, physical activity (self-reported frequency of exercise), and use of health services (self-reported general practitioner consultations or hospital visits). All outcomes were measured at the start of the trial (baseline before the intervention), immediately following the intervention (6 weeks after baseline), and 6 weeks after the intervention was finished (12 weeks after baseline). 

### Statistical Analysis

The study was powered to detect a difference of 2 points in the change over time (to the 12-week endpoint) of the WEMWBS score. Based on an estimated population mean score of 49.8 (from the Scottish Health Survey 2008) [20], and a standard deviation for mean change in WEMWBS scores over time of 9.84 [21], we required approximately 510 participants in each group with full data (for 2-sided type I error rate α = .05, power of 90%). Allowing for a high level of attrition (estimate 50%) as is common in fully automated Internet interventions, we aimed to recruit 2040 participants in total.

For the analysis of the primary endpoint and secondary endpoints where possible, (generalized) linear mixed models for repeated measures were fitted. These models appropriately account for the correlation between measurements from the same subject at different time points (baseline, 6-week follow-up, and 12-week follow-up). Models for each endpoint consisted of 3 effects: measurement occasion (time), intervention (MoodGYM or waiting-list control), and the interaction effect of time and intervention. Of primary interest was the intervention by time interaction effect. This effect informs whether the intervention type had a differential effect on the change over time in the two groups, thus answering the primary research hypothesis. Adjusted least squares means estimates and standard errors are presented for each endpoint and each model. An unstructured covariance matrix was used for modeling of correlations between repeated observations as this covariance matrix yielded the best fit among investigated structures for all endpoints.

Secondary endpoints which did not satisfy distributional assumptions for the repeated measures analysis were compared using paired *t* tests. These *t* tests were utilized to compare changes of outcome values between time points for MoodGYM and waiting-list control rather than absolute outcome values. Change scores fulfilled distributional assumptions of the *t* test where applied. Simple descriptive statistics (mean, median, standard deviation, range) were used to compare baseline characteristics of the two groups. The statistical analysis was conducted using the statistical software package SAS release 9.2 (SAS Institute, Inc, Cary, NC, USA). Ordinary linear mixed models were fitted using the MIXED procedure and the GLIMMIX was employed for fitting generalized linear mixed models. A 2-sided type I error rate of 5% was used throughout. Analyses were conducted on an intention-to-treat basis, including all participants in the groups to which they were randomized.

## Results

### Participation Rates

**Figure 2:**
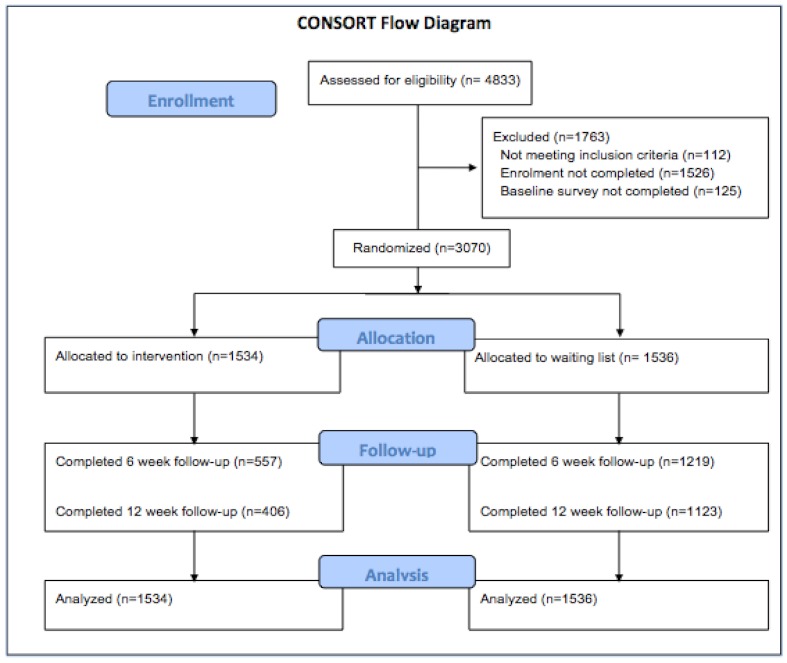
CONSORT flow diagram.

The trial flow diagram (Figure 2) shows participant recruitment and retention at baseline, 6-week follow-up, and 12-week follow-up. Over a 2-week period in September 2010, 8589 people accessed the URL; 4833 people completed the eligibility screening for the study and were sent invitation emails. Of these, 3070 returned the completed consent forms and baseline measures and were randomized into the study.

Attrition rate was high in this study. Total losses to follow-up were 50%, with participants in the intervention arm more likely to withdraw from the study. Attrition was 73.5% in the intervention arm and 26.9% in the control arm (risk ratio 2.76, 95% CI 2.53-3.02). No relationship between baseline characteristics and likelihood of withdrawal could be established. The WEMWBS score at baseline and posttest was slightly higher for participants retained in the trial until the end for both arms than for those participants who withdrew, but this difference was small and not statistically significant. A small number of participants (61 MoodGYM, 77 control) had missing observations at the 6-week follow-up, but provided responses at the later follow-up.

### Comparison of Baseline Characteristics

Table 1 summarizes the demographics and baseline characteristics of participants in the trial who were well balanced between the treatment groups. Most of participants were female (77.88%), in line with the general profile of the users of the NHS Choices portal that we used for recruitment, and 92.15% reported white ethnicity. The mean age was 41 years. Most participants were using the Internet daily and rated themselves as having either good or excellent Internet ability. More than half of the study participants had previously received treatment for a mental health problem.

**Table 1:** Demographic and baseline characteristics of trial participants (N=3070).

Variable		MoodGYM (n=1534)	Control (n=1536)
**Gender, n (%)**			
	Female	1179 (76.86)	1212 (78.91)
			
**Age (years), mean (SD)**		40.88 (12.96)	41.39 (13.05)
**Ethnicity, n (%)**			
	White	1408 (91.79)	1421 (92.51)
	Mixed	23 (1.50)	26 (1.69)
	Asian	33 (2.15)	30 (1.95)
	Black	26 (1.69)	16 (1.04)
	Other	29 (1.89)	31 (2.02)
	Invalid/missing	15 (0.98)	12 (0.78)
**Marital status, n (%)**			
	Married/cohabiting	723 (47.13)	711 (46.29)
	Divorced/separated	266 (17.34)	292 (19.01)
	Never married	545 (35.53)	533 (34.70)
**Employment status, n (%)**			
	Working	953 (62.13)	916 (59.64)
	Student	56 (3.65)	61 (3.97)
	Retired	93 (6.06)	101 (6.58)
	Looking after home/family	202 (13.17)	234 (15.23)
	Unemployed	174 (11.34)	181 (11.78)
	Other	56 (3.65)	43 (2.80)
**Smoking, n (%)**			
	Daily	218 (14.21)	214 (13.93)
	Occasionally	103 (6.71)	108 (7.03)
**Units of alcohol in past week, mean (SD)**		2.93 (3.88)	3.14 (4.16)
**Drug use in past week, n (%)**			
	Yes	53 (3.46)	40 (2.60)
**Internet use frequency, n (%)**			
	At least once a day	1361 (88.72)	1348 (87.76)
	Several times week	165 (10.76)	171 (11.13)
	Less than once a week	8 (0.52)	17 (1.11)
**Internet ability, n (%)** ^**a**^			
	Excellent	847 (55.22)	822 (53.52)
	Good	560 (36.51)	578 (37.63)
	Fair	123 (8.02)	127 (8.27)
	Poor	2 (0.13)	4 (0.26)
	Bad	2 (0.13)	1 (0.07)
**General health score, mean (SD)**		69.12 (21.10)	69.04 (20.40)
**Previous treatment of a mental health problem, n (%)**			
	Yes	877 (57.17)	843 (54.88)
**Previous CBT experience, n (%)**			
	Yes	326 (21.25)	321 (20.90)
**Previous Internet-based CBT, n (%)**			
	Yes	116 (7.56)	114 (7.42)
** Number of days in past week with > 30 minutes physical activity, n (%) **			
	0	418 (27.25)	408 (26.56)
	1	254 (16.56)	261 (16.99)
	2	287 (18.71)	281 (18.29)
	3	222 (14.47)	221 (14.39)
	4	134 (8.74)	107 (6.97)
	5	99 (6.45)	124 (8.07)
	6	36 (2.35)	48 (3.13)
	7	84 (5.48)	86 (5.60)

^a^ Control group responses n=1532.

#### Primary Endpoint Analysis

The primary research hypothesis of the trial was that MoodGYM improves well-being measured by WEMWBS at 6-week and 12-week follow-ups. Table 2 displays the adjusted WEMWBS score means on each measurement occasion. The difference at baseline, 6 weeks, and 12 weeks was –0.124 (95% CI –0.814 to 0.566), 2.542 (95% CI 1.693-3.390), and 2.876 (95% CI 1.933-3.819) points, respectively.

**Table 2:** Estimates of marginal means over balanced populations and standard errors for the Warwick-Edinburgh Mental Well-being Scale (WEMWBS).

Time point		WEMWBS scores	
		MoodGYM	Control
**Baseline**			
	Marginal mean	42.20	42.32
	Standard error	0.251	0.246
**6-week follow-up**			
	Marginal mean	44.46	41.92
	Standard error	0.343	0.265
**12-week follow-up**			
	Marginal mean	45.17	42.30
	Standard error	0.387	0.285

The results from a mixed model repeated measures analysis including time, intervention, and the interaction between them are given in the upper part of Table 3. The interaction effect (intervention × time point) is highly significant (*P*<.001), indicating that the intervention, MoodGYM, has a differential treatment effect compared with the control arm. A partition of the interaction effect to provide comparison of the two groups at each time point [22], indicated that there was no difference at baseline (*P*=.72) but that differences at 6 and 12 weeks were both highly significant (*P*<.001 in each case). Covariates were added to the model individually to determine those that had an influence on the model fit. All covariates that improved the model fit (in terms of the Akaike Information Criterion [AIC] using a likelihood ratio test) were included in the full model shown in Table 3. The overall model fit was significantly better than for the model excluding covariates (*P*<.001). The intervention by time interaction remained highly significant (*P*<.001). Although previous treatment for a mental health problem explained a significant amount of variation in the model, previous treatment did not have a significant impact on the change of WEMWBS scores over the study duration. There was no significant covariate by intervention interaction for any of the investigated covariates.

**Table 3:** Type III test of fixed effects for primary endpoint (WEMWBS).

Effect		*F* test (*df*)	*P*
**Basic model (AIC = 44,482)** ^**a**^			
	Intervention	23.74 (1,3068)	< .001
	Time point	25.68 (2,3301)	< .001
	Intervention × time point	33.87 (2,3301)	< .001
**Full model (AIC = 43,959)** ^**a**^			
	Intervention	31.08 (1,3057)	< .001
	Time point	25.44 (2,3299)	< .001
	Intervention × time point	33.51 (2,3299)	< .001
	Mental health service	197.04 (1,3057)	< .001
	Mental health service × time point	0.46 (2,3299)	.63
	Physical activity	16.00 (7,3057)	< .001
	Previous CBT use	19.92 (1,3057)	< .001
	Smoking	24.11 (2,3057)	< .001

^a^ AIC: Akaike Information Criterion.

To explore whether bias was introduced through systematic participant dropout, we undertook a completer analysis using the observed mean scores for those participants who completed all 3 investigations. Mean scores for those who adhered to the follow-up schedule were slightly higher on each occasion in both the MoodGYM and control arms than for the overall study population. However, mean WEMWBS scores at baseline for completers were only 0.42 and 0.13 points above the full study population means for MoodGYM and control groups, respectively, indicating that there was no systematic dropout of participants with lower baseline scores.

Given the high level of attrition, a sensitivity analysis with imputed missing values was conducted to evaluate the robustness of the effect of MoodGYM on mental well-being [23]. Missing values were imputed using the last observation carried forward (LOCF) procedure. This procedure is known to possess poor properties, underestimating variability and producing biased treatment effect estimates [24]. The LOCF procedure was only used here as a sensitivity analysis to investigate robustness given the high level of attrition. The estimated mean WEMWBS scores for the imputed dataset at 12-week follow-up were 43.34 and 42.24 for MoodGYM and control, respectively. Even under this highly conservative assumption, the interaction of intervention and time remained highly significant (*P*<.001).

#### Analysis of Secondary Endpoints

##### Depression and Anxiety

The results presented in Tables 4 and 5 show that MoodGYM and the waiting-list control had a significantly different effect on the CES-D score and GAD-7 score over time with participants in the intervention (MoodGYM) arm reporting a reduction in levels of depression and anxiety. The time by intervention interaction was highly significant (*P*<.001) for both endpoints. The differences at baseline, 6 weeks, and 12 weeks were –0.041 (95% CI –0.993 to 0.911), –2.793 (95% CI –3.947 to –1.640), and –3.365 (95% CI –4.621 to –2.110) points respectively for CES-D, and 0.212 (95% CI –0.074 to 0.758), –1.124 (95% CI –1.607 to –0.642), and –1.495 (95% CI –2.030 to –0.960) points for GAD-7.

**Table 4:** Estimates of marginal means over balanced populations and standard errors for Center for Epidemiologic Studies Depression scale (CES-D) endpoint.

Time point		CES-D scores	
		MoodGYM	Control
**Baseline**			
	Marginal mean	23.23	23.27
	Standard error	0.338	0.348
**6-week follow-up**			
	Marginal mean	20.38	23.17
	Standard error	0.469	0.356
**12-week follow-up**			
	Marginal mean	19.30	22.67
	Standard error	0.515	0.381

**Table 5:** Estimates of marginal means over balanced populations and standard errors for Generalized Anxiety Disorder 7-item scale (GAD-7) endpoint.

Time point		GAD-7 scores	
		MoodGYM	Control
**Baseline**			
	Marginal mean	8.80	8.46
	Standard error	0.151	0.149
**6-week follow-up**			
	Marginal mean	7.17	8.29
	Standard error	0.193	0.153
**12-week follow-up**			
	Marginal mean	6.60	8.10
	Standard error	0.221	0.161

##### Quality of Life, Physical Activity, and Health Service Use

The EQ-5D quality of life data were bimodal rendering an analysis using a linear mixed model impossible. Instead, *t* tests comparing the individual changes of EQ-5D scores between the two treatment arms were conducted. Change data were sufficiently normally distributed for the changes between baseline and the second follow-up measurement. There were no significant differences between arms for the change to 6 weeks (*P*=.78) or to 12 weeks (*P*=.42).

Physical activity was measured by using an ordered categorical variable (“In the past week, on how many days have you done a total of 30 minutes or more of physical activity, which was enough to raise your breathing rate”). Therefore, we used a mixed-effects proportional odds model to analyze this endpoint. This models the probability of being in category “*x* days of activity” or fewer days of activity for each of the 8 categories. Direct maximum likelihood estimation was used instead of the restricted maximum likelihood estimation employed in presented linear mixed models. The interaction term was significant indicating that the patterns of change of physical activity over time were significantly different between the two groups. Although significant, the effect (*F* test) was smaller than for the primary outcome and the secondary endpoints CES-D and GAD-7. The impact of time appears to be greater than the impact of treatment on this endpoint. Examination of the data suggests that the difference is explained by participants in the control group being more likely to report reduced activity at 12 weeks.

The number of general practitioner visits and hospital outpatient visits during the previous month were reported at baseline, 6 weeks, and 12 weeks and compared between the 2 groups. Repeated measures generalized linear mixed model for count data assuming a Poisson distribution were fitted for both secondary endpoints. The results showed that there was no significantly different effect between the groups on the mean number of GP visits over time (*P*=.30). There was also no differential effect between the MoodGYM and waiting-list control groups on the mean number of hospital attendances (*P*=.32).

#### Prespecified Subgroup Analyses

In order to investigate the consistency of the treatment effect, subgroup analyses based on age, gender, psychiatric history, previous use of CBT, level of anxiety, and level of depression were prespecified in the protocol. For each subgroup, a mixed model consisting of a time, group, and time × group effect was fitted. The results of these analyses are shown in Table 6. The treatment effect was very consistent across subgroups. Changes in the WEMWBS primary outcome remained significant for all subgroups except those aged under 26 years (change of 2.13 points, *P*=.22); however, the numbers in this subgroup were small as indicated by the wide confidence intervals. The 95% CI for participants over the age of 25 is entirely contained in the CI for those aged under 26 years. Thus, there is no evidence for a differential treatment effect between these two subgroups. Of note, the nondepressed and nonanxious subgroups of those with a CES-D score less than 16, or a GAD-7 score less than 10, both showed significant improvement in their WEMWBS well-being scores (*P*<.001).

**Table 6:** Subgroup analysis for primary endpoint, the Warwick-Edinburgh Mental Well-being Scale (WEMWBS) score.

Subgroup characteristic	Baseline			12-week follow-up				*F* (*df*)^a^	*P*
	MoodGYM mean (n)	Control mean (n)	Mean diff	MoodGYM mean (n)	Control mean (n)	Mean diff	95% CI		
Age < 26 years	41.93 (182)	41.51 (172)	0.42	45.63 (30)	43.50 (114)	2.13	–0.94, 5.20	1.50 (2,319)	.22
Age > 25 years	42.23 (1352)	42.42 (1364)	–0.19	45.13 (376)	42.17 (1009)	2.96	1.97, 3.95	33.40 (2,2978)	<.001
Female	42.29 (1179)	42.27 (1212)	0.02	44.95 (327)	42.15 (889)	2.80	1.76, 3.83	23.08 (2,2614)	<.001
Male	41.88 (355)	42.50 (324)	–0.62	45.97 (79)	42.80 (234)	3.17	0.97, 5.37	11.75 (2,683)	<.001
Psychiatric history	39.47 (877)	39.85 (843)	–0.38	42.48 (219)	39.59 (621)	2.89	1.62, 4.17	20.97 (2,1830)	<.001
No psychiatric history	45.83 (657)	45.32 (693)	0.51	48.74 (187)	45.59 (502)	3.15	1.83, 4.47	13.51 (2,1467)	<.001
Previous CBT	38.70 (326)	39.40 (321)	–0.70	41.00 (93)	38.74 (251)	2.26	0.37, 4.16	7.40 (2,750)	.007
No previous CBT	43.14 (1208)	43.09 (1215)	0.05	46.38 (313)	43.26 (872)	3.12	2.06, 4.19	28.18 (2,2547)	<.001
GAD-7 < 10	46.70 (915)	46.77 (934)	–0.07	48.77 (242)	46.00 (683)	2.78	1.67, 3.88	17.72 (2,2034)	<.001
GAD-7 > 9	35.53 (619)	35.42 (602)	0.11	39.90 (164)	36.60 (440)	3.30	1.83, 4.77	16.94 (2,1263)	<.001
CES-D<16	51.46 (506)	51.51 (514)	–0.05	51.99 (139)	49.72 (379)	2.28	0.85, 3.70	9.70 (2,1114)	<.001
CES-D>15	37.64 (1028)	37.70 (1022)	–0.06	41.76 (267)	38.57 (744)	3.18	2.09, 4.28	24.71 (2,2183)	<.001
CES-D>26	33.94 (601)	33.96 (596)	–0.02.	38.52 (150)	35.74 (437)	2.77	1.31, 4.24	15.82 (2,1265)	<.001

^a^ group × time.

#### Use of Intervention

**Figure 3:**
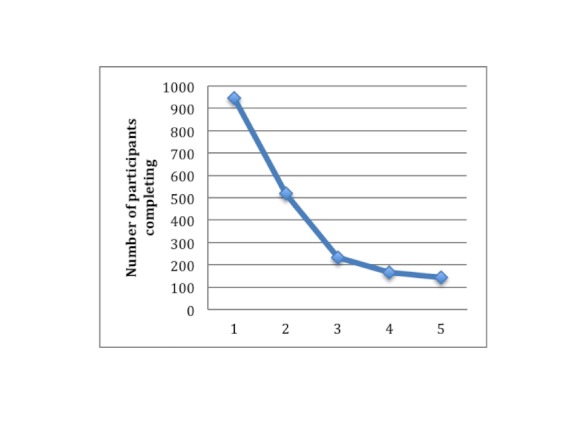
Number of completed modules by participants in intervention group.

Figure 3 shows the number of completed modules of the MoodGYM intervention by number of participants in the intervention arm.

**Figure 4:**
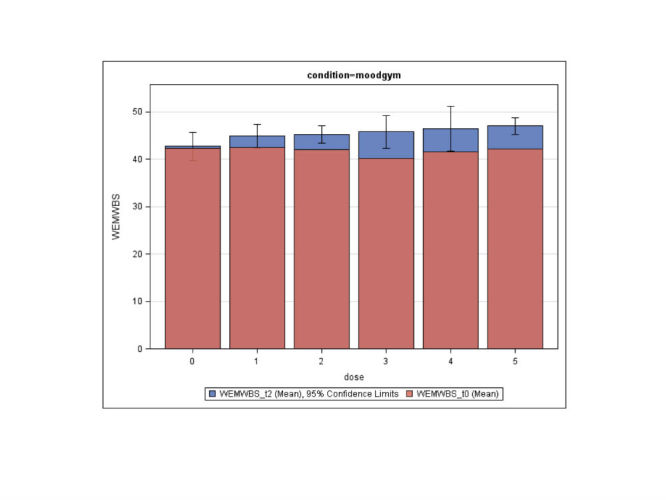
Mean Warwick-Edinburgh Mental Well-being Scale (WEMWBS) scores by number of completed weeks. Red is mean score at baseline and blue is mean score at 12-week follow-up.

A post hoc exploratory dose-response analysis to investigate the relationship between number of modules of the MoodGYM intervention completed and change in well-being, revealed that the change from baseline WEMWBS score to score at 12 weeks was significant for participants in the intervention group (“condition=moodgym”) who completed 2 or more modules (Figure 4).

#### Adverse Events

We received emails from 2 participants who indicated that they were suffering some level of mental distress that could possibly be related to the intervention. Neither was deemed to be a serious adverse event by the Trial Steering Committee, and both were reported to the ethics committee. In the first instance, a trial participant said that they no longer wished to continue with the trial having found one section of the intervention (on warpy thoughts) difficult to complete. In the second instance, a trial participant reported finding the intervention distressing to complete and asked to be withdrawn. Both participants were withdrawn immediately and given advice on seeking help from their primary care provider or from mental health services.

### Discussion

#### Main Findings

We successfully delivered a fully automated health promoting intervention to a large sample of the general population using the Internet. On average, those allocated to receive the intervention improved their mental well-being scores by almost 3 points on the WEMWBS scale over a 12-week period, whereas the scores for those in the waiting-list control group (who received no intervention) remained nearly unchanged. This effect was highly statistically significant (*P*<.001). The observed change of 2.876 points on the WEMWBS scale represents an effect size (Cohen’s *d*) of approximately 0.34. In a public health intervention designed to shift the whole distribution of mental well-being upwards in a population, such a difference can be important because a small difference per individual can bring a major benefit in population terms (as seen, for example, in public health interventions to reduce blood pressure). Analyses of secondary outcomes showed significant improvements (*P*<.001) in self-report measures of depression (CES-D) and anxiety (GAD-7). There were no significant differences in measures of quality of life (EQ-5D) or self-reported health service use. There was also a significant difference (*P*=.002) in self-reported physical activity at 12-week follow-up, explained by participants in the control group being more likely to report reduced activity. Our data on participant usage confirms high attrition rates and shows that a relatively low proportion of participants completed all 5 modules, and a post hoc dose-response analysis found statistically significant improvements in mental well-being (from baseline scores) in those completing 2 or more modules.

#### Limitations

Although we sought volunteers from the general population, the people who volunteered to take part in the research had relatively low initial mental well-being scores, which is not surprising given that we requested volunteers to take part in research to improve their mental well-being and the recruitment routes included advertisements placed on the mental health webpages of NHS Choices. The mean WEMWBS score for our participants was 42. The general population average (obtained from the Scottish Health Survey) is 49.8 (SD 8.3) [20]. Our study population also had relatively high mean scores on measures of depression (CES-D scale) and anxiety (GAD-7) scale, and a high level of previous treatment of a mental health problem, confirming that although our aim was to recruit volunteers from across the general population, an intervention for promoting mental well-being had particular salience for those with some level of mental health problems. This means we cannot be certain that a similar increase in well-being would be observed in a population with no prior mental health problems, although, importantly, our subgroup analyses showed that the treatment effect remained highly significant between the two arms of the trial within the subgroup of nondepressed participants and within the subgroup of participants with no previous treatment of mental health problems.

The trial was waiting-list controlled, so we cannot rule out the possibility of a placebo effect. We did not follow up participants beyond 3 months and further work on long-term effectiveness would be desirable. There was a low level of male participation in the trial, although the ratio of male to female participants was in line with the profile of users of the portal from which we recruited. There was a high level of dropout from the trial, particularly in the intervention arm. We tried to minimize dropout by incorporating a 48-hour period between passing eligibility screening and being accepted into the trial and by not randomizing until after all baseline measures had been completed. In this way, we hoped to recruit participants with some commitment to returning to the website and participating in the study. Most people who dropped out did not inform us, but simply stopped returning to the site or responding to emails. It is likely that more participants were retained in the control arm as they had an incentive to stay in (they were on the waiting list to receive the intervention), and the tasks they were required to complete during the trial (surveys at 6 weeks and 12 weeks) were less demanding than for the intervention group (intervention and surveys). Importantly, no systematic differences between those who dropped out and those who completed in either the intervention or the control groups could be identified, and there was no systematic dropout of participants with lower baseline scores. A sensitivity analysis that assumed that those who dropped out would have had no change in their well-being scores, showed that under this assumption the intervention would still have had a significant positive effect on mental well-being. Self-directed Internet interventions are known to have low rates of adherence [25], but this is potentially less of a problem in well-being promotion for the general population than for the treatment of mental illness because it does not raise ethical questions of inadequacy of treatment of a diagnosed health problem. Moreover, as a mental health promotion tool, the intervention can be delivered at very low marginal cost using minimal personnel resources so that it can be made freely available to all who wish to use it, in contrast to a therapist contact approach which would be neither feasible nor affordable for all.

#### Comparison With Other Studies

This was the first trial to evaluate the promotion of mental well-being using an Internet-based CBT approach. Previous trials of Internet-based CBT approaches have shown effectiveness in treating mild to moderate depression and in the prevention of depression [11]. A recent systematic review found 5 randomized controlled trials that used positive psychology interventions (PPI), some of which used cognitive-behavioral principles, delivered over the Internet and measured well-being as an outcome [26]. Three of the studies in the review targeted adults with depression [27-29]. Of the 2 studies that included general population samples, one used a strengths intervention (identifying and using your strengths) delivered to an Australian population recruited through online advertisements. This trial showed a significant improvement on 1 of 4 a priori well-being outcome measures (the Personal Well-being Index), but not on the other 3 [30]. The other trial tested an online resilience-training package for sales managers, also in Australia that found no improvement on the Authentic Happiness Index [31]. Both of these trials had high levels of attrition (83% and 41.5%, respectively) as found in our study. Both studies had far fewer participants than in the present study (160 and 53 participants, respectively). The study by Mitchell and colleagues [26] used an information-only placebo control group given online information about problem solving, whereas the study by Abbott and colleagues [31] used a waiting-list control group.

#### Conclusions

This study demonstrated that a low-cost, easily accessible, highly scalable, and self-directed intervention delivered in a fully automated fashion can be effective at improving mental well-being among regular Internet users recruited from the general population accessing a national health portal in England. Given the potential societal benefits of an increase in population well-being and the cost advantages of Internet-delivery with no practitioner contact, this could have major implications if accessed more widely. We have also demonstrated in this study that a national health portal provides a feasible and acceptable platform for the successful and rapid recruitment of participants into research. The trial procedures including consent and all baseline and follow-up measures were fully automated with implications for the future conduct and cost of trials with designs that could harness this.

Further work is needed to evaluate the effect of MoodGYM on mental well-being against a control website, to follow up participants more completely and for longer periods of time, and to target those who are not currently depressed to disaggregate the effect on mental well-being from the effect on depression and anxiety. This last aim could perhaps be achieved by recruiting participants from a nonhealth website. There is also a general need to further explore the relationship between intervention adherence and outcomes [32]. Intervention development could follow other investigators in this field and explore how to increase adherence, perhaps by examining user motivations to persist [33], and trialing alternate modes of delivery [34], which may include mobile health applications. Finally, there is also a need for rigorous evaluation of CBT-based approaches in comparison with other approaches that may improve well-being, such as positive psychology and mindfulness interventions.

## Multimedia Appendix 1

Completed CONSORT-EHEALTH checklist V.16 [35].

## Multimedia Appendix 2

Trial protocol as requested in CONSORT-EHEALTH [35].

## Multimedia Appendix 3

Trial information for participants as requested in CONSORT-EHEALTH [35].

## Abbreviations

AIC: Akaike Information Criterion
CBT: cognitive behavioral therapy
CCBT: computerized cognitive behavioral therapies
CES-D: Center for Epidemiologic Studies Depression scale
EQ-5D: EuroQol Group 5-Dimension Self-Report Questionnaire
GAD-7: Generalized Anxiety Disorder 7-item scale
LOCF: last observation carried forward
NHS: National Health Service
PPI: positive psychology interventions
WEMWBS: Warwick-Edinburgh Mental Well-being Scale
