# Supplementary material for: Effectiveness of a Web-Based Cognitive-Behavioral Tool to Improve Mental Well-Being in the General Population: Randomized Controlled Trial
Source: J Med Internet Res. 2013 Jan 9;15(1):e2. doi: 10.2196/jmir.2240 (PMC3636304; doi:10.2196/jmir.2240)
Supplement: Supplementary file 2 [file jmir_v15i1e2_app2.pdf]

Research protocol

The PsyWell study: A randomised controlled trial of an internet-based cognitive behaviour therapy intervention to improve psychological wellbeing
Version 5.0
25.1.2010

Author and lead investigator:
Dr John Powell
Associate Clinical Professor in Epidemiology and Public Health, Warwick Medical School, Gibbet Hill Road, Coventry CV4 7AL.

Co-investigators:
Dr Amanda Burls, University of Oxford
Professor Helen Christensen, Australian National University
Professor Kathy Griffiths, Australian National University
Dr Kylie Bennett, Australian National University
Anthony Bennett, Australian National University

Comments to: john.powell@warwick.ac.uk

Summary

This project is evaluating whether a self-directed training tool delivered via the internet can help people to improve their mental wellbeing. By mental wellbeing we mean a state of positive mental health where people are able to develop their potential, work productively and creatively, build strong and positive relationships with others, and contribute to their community. It is not just the absence of mental illness. Mental wellbeing is an important factor for issues such as social cohesion, social inclusion and prosperity. The tool we are evaluating uses a cognitive behavioural therapy (CBT) based approach. CBT is one approach which may be effective in promoting a positive state of mental wellbeing in the general population through encouraging more 'healthy' patterns of thinking and behaviour. We are choosing to deliver this intervention via the internet in a self-directed fashion. Many people now use the internet for various aspects of their healthcare.

We are evaluating this tool using a randomised controlled trial. We will ask visitors to the NHS Choices website whether they wish to take part in a trial and we will give them full information about it. They will of course be free to decline or to withdraw at any stage. If they choose to take part then half the participants (the 'intervention group') will be selected at random to get access to the CBT tool, and half the participants will be assigned to a 'waiting list group'. This waiting list group will still get access to the tool, but they will have to wait till the end of the trial (3 months) before they do. We will measure mental wellbeing and other issues using questionnaires. We will compare the change in scores in the intervention group with those in the waiting list group to see whether the tool does increase mental wellbeing.

1. Background

Mental wellbeing, or positive mental health, is a complex construct that includes both affect and psychological functioning. Two definitions are helpful in summarising this construct. First, the World Health Organisation's (WHO) definition of mental health:

> *". . .a state of wellbeing in which the individual realizes his or her own abilities, can cope with the normal stresses of life, can work productively and fruitfully, and is able to make a contribution to his or her own community." (WHO, 2005) [1]*

Second, the Foresight Report on Mental Capital and Wellbeing produced for the Government Office for Science used this definition:

> *"This is a dynamic state, in which the individual is able to develop their potential, work productively and creatively, build strong and positive relationships with others, and contribute to their community. It is enhanced when an individual is able to fulfil their personal and social goals and achieve a sense of purpose in society." (Foresight Mental Capital and Wellbeing Project, 2008).[2]*

There is now worldwide interest in the promotion of mental wellbeing with recognition of the importance of its influence on behaviour, social cohesion, social inclusion and prosperity. Indeed, measures of wellbeing are now being adopted as key economic indicators, alongside GDP (Report by the Commission on the Measurement of Economic Performance and Social Progress, 2009).[3]

We are therefore interested in interventions that can promote mental wellbeing and positive mental health. Cognitive behavioural therapy (CBT) is one such approach. According to the National Institute for Health and Clinical Excellence (NICE):

> *"CBT is a generic term that refers to the pragmatic combination of concepts and techniques from cognitive therapy and behavioural therapy. Both of these use structured approaches based on the assumption that prior learning is currently having maladaptive consequences. The purpose of therapy is to reduce distress or unwanted behaviour by undoing this learning or by providing new, more adaptive learning experiences."*

Computerised CBT has been demonstrated to be an effective treatment for helping people with anxiety and depression [4] and there is emerging evidence that computerised training might be useful, not only for treating those with mental health problems, but also for preventing the onset of problems and for promoting a positive state of wellbeing in the general population through encouraging healthy cognitive patterns. [5, 6]

The internet is playing an increasingly important role in healthcare. It is seen as providing the means to deliver more informed, empowered citizens who are better able to manage their own health; and to provide interventions for modifying lifestyle risk factors, all at low marginal cost using little in personnel resource. The NHS itself is beginning to harness the potential of the internet and related technologies for health care and health promotion through initiatives such as NHS Choices. Since therapist-led CBT, even if effective for promoting wellbeing, would not be logistically or economically feasible to deliver on the wide scale that would be required for the promotion of mental wellbeing in the general population, we propose to investigate the effectiveness of self-enrolled online automated CBT training. The increasing availability and decreased cost of home broadband connections, the connected nature of the modern workplace, and a general increase in "e-literacy" mean that if effective, this intervention would be able to be accessed by millions of UK citizens at a minimal cost.

We propose to use an online generic CBT training tool, MoodGYM. This was originally developed as a tool for young adults to teach them about stress and mental health problems and to introduce cognitive-behavioural principles in order to prevent the development of mental health problems. Since then it has undergone modifications for use in adults of all ages

and the current average age of users is 36 years. It has already been shown to be safe and effective for the treatment of some mental health problems such as mild to moderate anxiety and depression. [7, 8, 9] MoodGYM consists of five interactive modules which use flashed diagrams and online exercises, to teach cognitive behavioural principles. It demonstrates the relationship between thoughts and emotions, examines issues related to stress and to relationships, and teaches relaxation and meditation techniques. It also includes sections on managing relationships and problem solving. Participants are encouraged to work their way through each of the modules. The site interactively provides feedback on mood levels. MoodGYM is currently freely available via the Australian National University website.

2. Aim and objectives

2.1. Aim

To use the NHS Choices platform to evaluate an intervention to improve the mental wellbeing of the general population using a randomised controlled trial

2.2. Objectives

- To test whether a self-delivered online CBT can promote mental wellbeing in the general population by undertaking a pragmatic online randomised controlled trial of the validated online CBT package MoodGYM and measuring its effect on wellbeing and measures of mood.
- To explore the feasibility of using the internet in general, and the NHS Choices platform in particular, as a method of delivering fast, safe and acceptable interventions to those who want them.

3. Methods

3.1. Design

A pragmatic randomised controlled trial with two arms: intervention and waiting-list control. Since the primary purpose of this study is to identify a safe, effective and acceptable

intervention that can be made available freely online for unrestricted use by any member of the public who wishes to improve their mental wellbeing, it is important that the trial design reflects the intended use as far as possible and is therefore pragmatic in design. A pragmatic trial aims to replicate the situation in which the intervention might be used if shown to be effective.

We have chosen a waiting-list control so that those participants in the control arm will be eligible to receive the intervention, although they will have to wait 3 months before accessing it. The choice of waiting list also reflects the pragmatic design of the trial, rather than using an attention placebo or 'treatment as usual' (there is not treatment as usual for mental wellbeing). During the intervention the control participants will not receive any specific intervention. In common with the participants in the intervention arm, control participants will be able to access general information pages on mental wellbeing on the NHS Choices site, but as a waiting list control they will receive no specific intervention over and above this.

Within the trial, in order to understand more about how and why the tool is actually used (or not used) in practice by the participants in the intervention arm, we will undertake a small qualitative interview study with 20 volunteer participants.

3.2. Population

Any adult willing to take part in an internet-based trial of a CBT-based intervention to improve mental wellbeing.

Inclusion criteria:

- Adults aged 18 or over
- Located in England
- Able to read and write English
- Have access to the internet in order to use the intervention
- Have an email address where they can be contacted

- Give informed consent

Exclusion criteria:

- Aged under 18
- Located outside England
- Unable to read and write English
- Unable to access the intervention
- No email address provided
- Email address has already been registered as a trial participant
- No consent given

Considerable consideration was given to the inclusion and exclusion criteria, with the aim of having as broad an inclusion as possible given the need for a pragmatic trial. Adults were chosen for two main reasons: firstly we felt it was preferable to establish that the intervention was effective in adults before trialling it on children or adolescents; secondly, it made the issue of informed consent more straightforward as it did not require getting the consent of a parent or guardian.

Location in England will be determined by self-report (drop down menu).

3.3. Intervention

A UK-adapted version of MoodGYM which will be accessible through a trial portal page on the NHS Choices website (participants will log into the system using an anonymous username and password).

MoodGYM is a 5 week program comprising of 5 interactive modules, a series of quizzes and 29 exercises. The modules provide cognitive therapy (cognitive restructuring), behavioural methods for overcoming dysfunctional thinking, relaxation, problem solving, assertiveness and

self-esteem training, and strategies for coping with relationship breakup. The 'quizzes' include a series of anxiety and depression scales (that permit the user to track the change in their symptoms across modules) and a range of other measures (such as quizzes which assist the user to understand their particular profile of thinking patterns or to identify their preferred and actual pleasant events profile). Feedback on the quizzes is based on normative data collected from large scale epidemiological surveys. Users can consult their workbook containing completed exercises at any time. At the end of each module, the user can, if they wish, print out a summary of their session, including the level of their depressive and anxiety symptoms, their scores on other tasks, their goals and achievements. This summary is written in a format that is intended to be taken to the person's health practitioner should this be deemed appropriate by the user. The user is provided with a personalised certificate of completion at the end of the five modules.

Participants in the intervention arm will receive weekly email reminders to log into the trial portal where they can access the intervention.

MoodGYM is an Australian package and qualitative work with previous UK users suggests that some of the wording will need to be adapted to the UK context for maximum acceptability. We would do this and provide the adapted package in the pilot phase in order to validate the culturally adapted version prior to launching the full trial.

The MoodGYM intervention will be delivered from the servers of the Australian National University, where the tool was developed, but from the perspective of the user it will be seen as a trial intervention on the NHS Choices platform.

3.4. Comparator

The control arm will be a waiting list control. Participants in both arms of the trial will be reminded (by email) of the general information on wellbeing provided on the NHS Choices website. They will be given full access to the intervention after a wait of 3 months. They will be notified of their access by email.

We will aim to minimise contamination (control participants accessing the intervention) by having individual password protected access to the intervention, and by not branding the intervention as MoodGYM in the information sheets and consent procedures (so control participants do not know what the intervention name is).

3.5. Outcomes

Primary outcome:

The primary outcome measure of the study is mental wellbeing. This will be measured using the 14-item Warwick-Edinburgh Mental Wellbeing Scale (WEMWBS) [10] as this instrument has been validated for the UK population. (See Appendix 1: outcome measures). A recent study has demonstrated its sensitivity to change before and after an intervention.[11]

Secondary outcomes:

- Depression - measured using CES-D, a depression inventory that has been validated for use via the internet. (Appendix 1).
- Anxiety - measured using the GAD-7, also validated for online use. (Appendix 1).
- Quality of Life - measured using the EQ5D, also validated for online use. (Appendix 1).
- Physical activity. (Appendix 1).
- Service use. (Appendix 1).

Measurements will be taken at baseline, after completion of the MoodGYM course (six weeks) and at three months (12 weeks).

3.6. Other Measurements

- Basic demographic data (age, sex, where they live, educational level, smoking status, ethnicity, marital status, etc).

- Access and use of MoodGYM and other internet interventions.
- Current physical activity levels.
- Previous psychiatric history (self-report of diagnosis received from a medical practitioner).
- Alcohol and drug consumption.
- Internet use

Measurements will be taken at baseline, after completion of the MoodGYM course (six weeks) and at three months (12 weeks) .

3.7. Identification and recruitment

Participants will be self-recruited over the internet from an advert placed on the NHS Choices website. This advert will offer participants the opportunity to take part in a mental fitness trial, with the aim of promoting wellbeing. It will not be advertised in any way as a therapy for people who are ill. Interested participants will directed to the trial URL where they will receive full details of the study including participant information sheet, frequently asked questions and contact details of the lead investigator (Powell at Warwick University) in order to ask further questions if necessary. These materials will be written by the research team. If visitors wish to participate in the trial they will complete online a screening questionnaire which will assess participants' eligibility for participation in the trial according to the inclusion criteria. If ineligible they will be thanked for their interest, informed of their ineligibility and directed to the Healthy Living pages on NHS Choices. If eligible they will be asked to provide their email address and set up a username and password. They will be asked to consider their participation in the trial for a period of 48 hours and informed that after 48 hours they will receive an email inviting them to log on to the trial portal and provide consent via a secure web form. Internet-based intervention trials often suffer high levels of attrition and this 48 hour period will not only give participants time to consider their participation in research, but will also serve as a way of reducing attrition (i.e. participants will need to demonstrate some level of commitment to returning to the site in order to take part in the trial), thereby decreasing bias in the study.

This model of providing consent online is being successfully used in a current UK trial of MoodGYM.[12] Participants who consent to participate in the trial will then be directed to the baseline demographic and mental health questionnaires. If they complete these baseline measures they will immediately be randomised and given access to the intervention (intervention arm) or informed of their allocation to waiting list (control arm).

For the qualitative interview study with 20 participants in the intervention arm, we will seek volunteers at the time of final follow-up, using a recruitment question in the final questionnaire. Volunteers will be required to give consent and will interviewed over the telephone, and audio-recorded.

3.8. Randomisation

We will use a customised randomisation facility which is currently being used in a variety of online trials in Australia, run by the Australian National University. The procedure allows for full replication and can accommodate stratification and specification of block sizes, although in this trial we will use simple randomisation to intervention or control in a ratio of 1:1.

Once randomised, participants in the active arm will immediately be provided with the first week of the intervention, plus information to access the pages on Healthy Living at NHS Choices. Individuals in the control arm will be given equivalent information about accessing the general information pages on Healthy living on NHS Choices and informed that they will receive the intervention after a period of 12 weeks (waiting list control group). Both groups will be informed about the follow-up measurements required at 6 weeks and 12 weeks.

3.9. Reminders

Participants in the intervention group will receive a weekly email reminder which contains the URL for the trial portal. They will also receive an extra email reminder if they have not logged in to the intervention for a week. All participants will receive email reminders at 6 weeks, and 12 weeks to complete the outcome measures. These automated reminders will be sent from the MoodGYM team.

The trial flow is summarised in Appendix 2.

3.10. Statistical analysis

Analysis and reporting of results will conform to CONSORT guidelines, as follows:

- Trial flow will be reported using a CONSORT diagram.
- Any deviations from the protocol will be reported, with reasons.
- We will report dates of recruitment and follow-up.
- We will report baseline characteristics of both groups (intervention and control).
- Analyses will be on an intention to treat basis, and the number of included participants will be clearly stated.
- For each primary and secondary outcome measure, a summary of results for each group will be reported, together with the estimated effect size and its precision.
- We will report any other analyses performed, including subgroup analyses and adjusted analyses.
- We will report any adverse events.

The primary outcome will be changes from baseline on the WEMWBS mental wellbeing scale, comparing the repeated measures (6 weeks, and 12 weeks) between the two groups. We will use mixed-model repeated-measures ANOVA with measurement occasion as a within-groups factor and intervention as a between-groups factor. We will compare changes from baseline in each group at 6-weeks post-test, and 12 week follow-up.

Secondary outcomes will be changes from baseline for anxiety and depression scores and quality of life measurement.

For all outcomes we will investigate interactions with age, sex, baseline mental health scores, and concordance with the intervention.

Additional analyses will include descriptive statistics to characterise participants in terms of baseline characteristics; CONSORT diagram of the flow of participants through the trial, and proportion who completed each stage to trial entry; and use of intervention.

The baseline data of those who entered the trial but did not complete follow up according to the trial protocol, and those who did, will be compared to determine how representative the trial results are and to investigate any potential threats to validity from differential losses to follow up in each arm.

Concordance with the intervention will be reported (number of modules completed).

We will measure and report any level of contamination (that is, participants in the control arm accessing the same or similar intervention to participants in the intervention arm).

The investigators undertaking analysis will be blind to the allocation of intervention or control.

3.11. Sample size

Sample size has been calculated for a comparison of the mean change in WEMWBS scores from baseline to final follow-up. The population mean score for WEMWBS (from the Scottish Health Survey 2008) is 50. In the recent study that demonstrated change over time for WEMWBS following an intervention, the standard deviation for mean change in WEMWBS scores over time was 9.84. In order to reliably detect a change of 2 points on the WEMWBS score (i.e. to 48 or to 52 for an individual with a score of 50), we need 510 participants in each group with full data (with alpha=0.05, power of 90%). Allowing for a high level of attrition (estimate 50%), we wish to recruit 2040 participants in total. This 50% estimate is based on the results of a cohort study of MoodGYM users which found that 1503 participants of 2909 (51.7%) completed at least one module,[13] and on a systematic review of adherence to internet interventions for anxiety and depression which found that completion of protocol rates ranged from 43% to 99%.[14] Given the fully automated nature of our self-directed intervention we are assuming an adherence rate at the low end of this range.

Once the requisite number of participants has been recruited, recruitment will be closed.

3.12. Qualitative analysis

The transcripts from the qualitative interviews with 20 participants in the intervention arm will be analysed thematically using the Framework Approach described by Ritchie and Spencer. This is a grounded method for analysing qualitative data consisting of familiarization with the data, identification of a thematic framework, indexing, charting, and mapping and interpretation. It provides a systematic and comprehensive way of analysing interview transcripts.[15]

3.13. Support to participants

Email will be the main support for technical or other problems.

There will be a link to FAQs on the trial website.

The email address, address and telephone number of the trial team (based at Warwick University) will be available on the website, and automated emails will include statements informing participants to reply to the email or to telephone or write to the trial team if they are having any problems accessing the website, or if they wish to withdraw from the study

3.14. Public Involvement

Once approval in principle has been agreed for this trial, we will recruit members of the general population to the advisory group to give the consumer perspective and be involved in the further development and running of this trial.

Consumers will be compensated for any expenses involved in their input to the trial development but they will not be paid for their time.

3.15. Ethical considerations

Ethics approval will be obtained from NHS MREC.

All participants will be required to give consent to take part in the trial and for their data to be used solely for the purposes of the trial. All data gathered in the trial will be stored electronically on secure servers and accessed via password protected computers. All data will relate to participant ID numbers only and will not be directly linked to personally identifiable information. The 'key' to the participant ID numbers will be stored separately.

Trial participants will be able to drop out of the intervention at any time without giving a reason. Although they will be encouraged to complete the outcome questionnaires to enable an intention to treat analysis to be undertaken, they will of course be free not to do so.

Participants in the comparator arm will be given access to the intervention after 3 months (waiting list control) so no participant will be denied access.

Members of the public whose scores on the depression scale suggest an important baseline mental health problem will be advised by email to see their GP or seek other NHS mental health support. We will use a cut-off of 26 for the CES-D depression scale (suggests need for investigation of possible major depression [16]). They will not be excluded from taking part in this trial to promote mental wellbeing. While it is ethically important to provide advice to seek help whether or not such people are included or excluded from the trial, we do not think it is necessary on ethical grounds to exclude them as the intervention has already been shown to be of some benefit in such conditions (and to our knowledge) not cause harm.[8, 9]

Participants whose scores on subsequent tests suggest the development of a mental health problem will be advised to see their GP or seek other NHS mental health support.

All responses will be kept anonymous and will be encrypted and kept in a secure way that meets current trial standards.

3.16. Quality Assurance

The trial will be registered on the International Standard Randomised Controlled Trial Number Register (http://www.controlled-trials.com/). This provides a documented record of the

primary outcome and proposed analysis to prevent post hoc hypothesis testing being open to over-interpretation.

A trial advisory committee will be established, with an independent chair and representation from the Department of Health, together with the lead researchers. One of the chief responsibilities of this group, aside from overseeing general progress, will be to monitor the trial for any adverse events or suggestions of harm occurring to participants. The group will have access to all qualitative feedback plus early sight of the quantitative results. The group will also assess whether emerging results are of relevance to current trials in progress and will disseminate the findings to these trials accordingly.

The trial protocol has been discussed with Warwick Clinical Trials Unit and has undergone internal and external peer review.

The trial will be subject to a pilot phase before full launch.

References

1. World Health Organization. (2005). Promoting Mental Health: Concepts, Emerging evidence, Practice: A report of the World Health Organization, Department of Mental Health and Substance Abuse in collaboration with the Victorian Health Promotion Foundation and the University of Melbourne. World Health Organization. Geneva.
2. Jenkins, R., Meltzer, H., Jones, P.B., Brugha, T., Bebbington, P., Farrell, M., Crepaz-Keay, D. And Knapp, M. (2008) Foresight Mental Capital and Wellbeing Project. Mental health: Future challenges.The Government Office for Science, London.
3. Stiglitz J. (2009) Commission on the Measurement of Economic Performance and Social Progress.
4. Kaltenthaler E, Parry G, Beverley C, Ferriter M. (2008). Computerised cognitive-behavioural therapy for depression: systematic review. Br J Psychiatry. 193(3):181-4.
5. Seligman, M.E.P., Steen, T.A., Park, N., & Peterson, C. (2005). Positive psychology progress: Empirical validation of interventions. American Psychologist, 60:410–421.
6. Mitchell J, Stanimirovic R, Klein B, Vella-Brodrick d. (2009) A randomised controlled trial of a self-guided internet intervention promoting well-being . Computers in Human Behavior. 25(3):749-760.
7. Christensen, H., Griffiths, K., Korten, A., Brittliffe, K. & Groves, C. (2004) A comparison of changes in anxiety and depression symptoms of spontaneous users and trial participants of a cognitive behaviour therapy website. Journal of Medical Internet Research 6(4):1-10.
8. Christensen, H., Griffiths, K. & Jorm, A. F. (2004) Delivering depression interventions using the internet: Positive results from a large randomised controlled trial. British Medical Journal 328:1-5.
9. Mackinnon, A., Griffiths, K. & Christensen, H. (2008) Comparative randomised trial of online cognitive - behavioural therapy and an information website for depression: 12-month outcomes. British Journal of Psychiatry. 192:130-134.
10. Tennant R, Hiller L, Fishwick R, Platt S, Joseph S, Weich S, Parkinson J, Secker J, Stewart-Brown S. (2007) The Warwick-Edinburgh Mental Wellbeing Scale (WEMWBS): development and UK validation. Health and Quality of Life Outcomes 5:63

11. Lindsay G, Band S, Cullen MA, Cullen S, Strand S, Hasluck C, Evans R, and Stewart-Brown S. (2009). Parenting Early Intervention Pathfinder Evaluation. Department for Children, Schools and Families. Research Report DCSF-RW054.
12. Full title of study: Computerised CBT for common mental disorders: a workplace RCT. NRES REC reference number: 08/H0401/91. Chief Investigator: Professor Justine Schneider, University of Nottingham. Study details at http://public.ukcrn.org.uk/search/StudyDetail.aspx?StudyID=6027
13. Christensen H, Griffiths KM, Korten A. (2002) Web-based Cognitive Behavior Therapy: Analysis of Site Usage and Changes in Depression and Anxiety Scores. J Med Internet Res 4(1):e3
14. Christensen H, Griffiths KM, Farrer L. (2009) Adherence in Internet Interventions for Anxiety and Depression: Systematic Review. J Med Internet Res 11(2):e13
15. Ritchie J, Spencer L. (1993) Qualitative data analysis for applied policy research. Pages 173-194. In: Bryman A, Burgess R, eds. Analysing qualitative data. London: Routledge.
16. Zich, J. M., C. C. Attkisson, et al. (1990). "Screening for depression in primary care clinics: the CES-D and the BDI." Int J Psychiatry Med 20(3): 259-77.

Appendix 1: outcome measures

1. Primary outcome: 14-item Warwick-Edinburgh Mental Wellbeing Scale (WEMWBS) (Tennant R, Hiller L, Fishwick R, Platt S, Joseph S, Weich S, Parkinson J, Secker J, Stewart-Brown S. (2007) The Warwick-Edinburgh Mental Wellbeing Scale (WEMWBS): development and UK validation. Health and Quality of Life Outcomes 5:63)

The Warwick-Edinburgh Mental Well-being Scale (WEMWBS)

Below are some statements about feelings and thoughts.

Please tick the box that best describes your experience of each over the last 2 weeks

STATEMENTS	None of the time	Rarely	Some of the time	Often	All of the time
I've been feeling optimistic about the future	1	2	3	4	5
I've been feeling useful	1	2	3	4	5
I've been feeling relaxed	1	2	3	4	5
I've been feeling interested in other people	1	2	3	4	5
I've had energy to spare	1	2	3	4	5
I've been dealing with problems well	1	2	3	4	5
I've been thinking clearly	1	2	3	4	5
I've been feeling good about myself	1	2	3	4	5
I've been feeling close to other people	1	2	3	4	5
I've been feeling confident	1	2	3	4	5
I've been able to make up my own mind about things	1	2	3	4	5
I've been feeling loved	1	2	3	4	5
I've been interested in new things	1	2	3	4	5
I've been feeling cheerful	1	2	3	4	5

"Warwick Edinburgh Mental Well-Being Scale (WEMWBS)
© NHS Health Scotland, University of Warwick and University of Edinburgh, 2006, all rights reserved."

2. CES-D (Radloff, L.S. (1977) 'The CES-D scale: A self report depression scale for research in the general population'. Applied Psychological Measurement 1: 385-401.)

Center for Epidemiologic Studies Depression Scale (CES-D), NIMH

Below is a list of the ways you might have felt or behaved. Please tell me how often you have felt this way during the past week.

	During the Past Week			
	Rarely or none of the time (less than 1 day)	Some or a little of the time (1-2 days)	Occasionally or a moderate amount of time (3-4 days)	Most or all of the time (5-7 days)
1. I was bothered by things that usually don't bother me.	☐	☐	☐	☐
2. I did not feel like eating; my appetite was poor.	☐	☐	☐	☐
3. I felt that I could not shake off the blues even with help from my family or friends.	☐	☐	☐	☐
4. I felt I was just as good as other people.	☐	☐	☐	☐
5. I had trouble keeping my mind on what I was doing.	☐	☐	☐	☐
6. I felt depressed.	☐	☐	☐	☐
7. I felt that everything I did was an effort.	☐	☐	☐	☐
8. I felt hopeful about the future.	☐	☐	☐	☐
9. I thought my life had been a failure.	☐	☐	☐	☐
10. I felt fearful.	☐	☐	☐	☐
11. My sleep was restless.	☐	☐	☐	☐
12. I was happy.	☐	☐	☐	☐
13. I talked less than usual.	☐	☐	☐	☐
14. I felt lonely.	☐	☐	☐	☐
15. People were unfriendly.	☐	☐	☐	☐
16. I enjoyed life.	☐	☐	☐	☐
17. I had crying spells.	☐	☐	☐	☐
18. I felt sad.	☐	☐	☐	☐
19. I felt that people dislike me.	☐	☐	☐	☐
20. I could not get "going."	☐	☐	☐	☐

SCORING: zero for answers in the first column, 1 for answers in the second column, 2 for answers in the third column, 3 for answers in the fourth column. The scoring of positive items is reversed. Possible range of scores is zero to 60, with the higher scores indicating the presence of more symptomatology.

3. GAD-7 (Spitzer RL et al. A brief measure for assessing generalized anxiety disorder: The GAD-7. Arch Intern Med 2006;166:1092-1097).

How often during the past 2 weeks have you felt bothered by:

1. Feeling nervous, anxious, or on edge?
   0 = not at all
   1 = several days
   2 = more than half the days
   3 = nearly everyday

2. Not being able to stop or control worrying?
   0 = not at all
   1 = several days
   2 = more than half the days
   3 = nearly everyday

3. Worrying too much about different things?
   0 = not at all
   1 = several days
   2 = more than half the days
   3 = nearly everyday

4. Trouble relaxing?
   0 = not at all
   1 = several days
   2 = more than half the days
   3 = nearly everyday

5. Being so restless that it is hard to sit still?
   0 = not at all
   1 = several days
   2 = more than half the days
   3 = nearly everyday

6. Becoming easily annoyed or irritable?
   0 = not at all
   1 = several days
   2 = more than half the days
   3 = nearly everyday

7. Feeling afraid as if something awful might happen?
   0 = not at all
   1 = several days
   2 = more than half the days
   3 = nearly everyday

4. EQ-5D (Rabin R, de Charro F. EQ-5D: a measure of health status from the EuroQol Group. Ann Med. 2001 Jul;33(5):337-43.)

By placing a tick in one box in each group below, please indicate which statements best describe your own health state today.

Mobility

I have no problems in walking about ❑

I have some problems in walking about ❑

I am confined to bed ❑

Self-Care

I have no problems with self-care ❑

I have some problems washing or dressing myself ❑

I am unable to wash or dress myself ❑

Usual Activities (e.g. work, study, housework, family or leisure activities)

I have no problems with performing my usual activities ❑

I have some problems with performing my usual activities ❑

I am unable to perform my usual activities ❑

Pain/Discomfort

I have no pain or discomfort ❑

I have moderate pain or discomfort ❑

I have extreme pain or discomfort ❑

Anxiety/Depression

I am not anxious or depressed ❑

I am moderately anxious or depressed ❑

I am extremely anxious or depressed ❑

To help people say how good or bad a health state is, we have drawn a scale (rather like a thermometer) on which the best state you can imagine is marked 100 and the worst state you can imagine is marked 0.

We would like you to indicate on this scale how good or bad your own health is today, in your opinion. Please do this by drawing a line from the box below to whichever point on the scale indicates how good or bad your health state is today.

Your own health state today

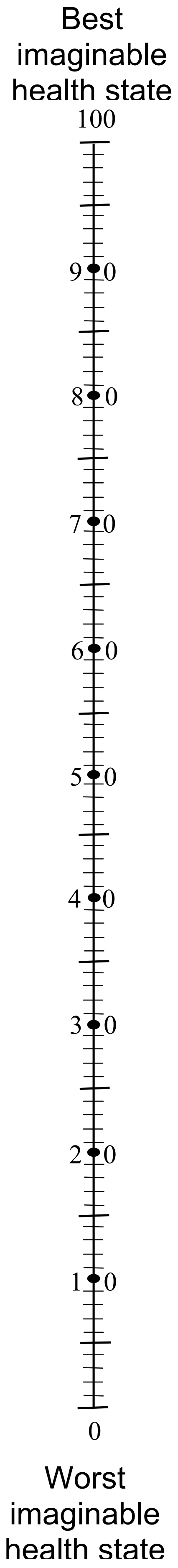

5. Single-item measure of physical activity (Milton,K., Bull,F.C. & Bauman,A. Reliability and Validity Testing of a Single-Item Physical Activity Measure. British Journal of Sports Medicine. *In press.* Accepted 3 December 2009.)

In the **past week**, on how many days have you done a total of **30 minutes or more** of physical activity, which was enough to raise your breathing rate?
This may include sport, exercise, and brisk walking or cycling for recreation or to get to and from places, but should not include housework or physical activity that is part of your job.

☐ 0 ☐ 1 ☐ 2 ☐ 3 ☐ 4 ☐ 5 ☐ 6 ☐ 7

6. Service use questions (adapted from questions on health service use in the General Household Survey, carried out by the Social Survey Division of the Office for National Statistics http://www.statistics.gov.uk/ssd/surveys/general_household_survey.asp)

Please answer the following questions about your use of health services.

In the last month:

How many times have you seen a General Practitioner (for any reason)? [drop down menu]

How many times have you been seen as a hospital outpatient (for any reason)? [drop down menu]

How many times have you been admitted to hospital as a hospital inpatient (for any reason)? [drop down menu]
