# Supplementary material for: Effectiveness of a Web-Based Cognitive-Behavioral Tool to Improve Mental Well-Being in the General Population: Randomized Controlled Trial
Source: J Med Internet Res. 2013 Jan 9;15(1):e2. doi: 10.2196/jmir.2240 (PMC3636304; doi:10.2196/jmir.2240)
Supplement: Supplementary file 3 [file jmir_v15i1e2_app3.pdf]

Psywell study: information page v 1.2

Would you like to take part in a research study to see if a training programme on a website can improve your mental wellbeing?

NHS Choices is looking for volunteers who live in England to take part in a research study to test an online training tool. The project is called the Psywell study. It is being run by researchers based at the University of Warwick in the UK and at the Australian National University.

The website training programme offers the opportunity for people to learn new skills in how to think and how to behave. It aims to help people to identify unhelpful patterns of thinking or behaviour, and then learn how to change them.

If you might be interested in taking part then please read the information on this page. Once you have read it please use the buttons at the foot of the page to tell us whether or not you want to volunteer.

Information about the research:

We have provided answers to the following frequently asked questions:

- What is the purpose of the study?
- Do I need to have a mental health problem?
- Do I have to take part?
- What will happen to me if I volunteer?
- What do I have to do if I take part in the research?
- What is the website that is being tested?
- What are the alternatives for treatment?
- What are the side effects of any treatment received when taking part?
- What are the possible disadvantages and risks of taking part?
- What are the possible benefits of taking part?
- What happens when the research trial stops?
- What if there is a problem?
- Will my taking part in this trial be kept confidential?
- What will happen if I don’t want to carry on with the study?
- What will happen to the results of the study?
- Who is organising and funding the research?
- Who has reviewed the study?

What is the purpose of the study?

The purpose of the study is to find out whether a website training programme can improve a person’s mental (psychological) wellbeing. The website training programme offers the opportunity

for people to learn new skills in how to think and how to behave. It aims to help people to identify unhelpful patterns of thinking or behaviour, and then helps them learn how to change these.

[back to top]

Do I need to have a mental health problem?

No. This study is testing whether an internet programme can help everybody improve their mental wellbeing.

[back to top]

Do I have to take part?

No. It is up to you whether you want to take part or not. You can say no now, or you can withdraw at a later date if you change your mind. If you decide not to take part, or if you change your mind later it will have no effect on any care you receive from the NHS.

[back to top]

What will happen to me if I volunteer?

We will ask you for some details about your age and where you live to see whether you are eligible to take part. We only want volunteers who are aged 18 or over and who live in England. If you are eligible then we will ask you to give us your email address so we can contact you again in two days time. The reason for the two day delay is so that you have time to think about whether or not you really do want to take part in this research.

When we email you we will ask you to visit a website where you will be asked to confirm whether or not you still want to take part. If you do we will then ask you to fill in a questionnaire which asks questions about yourself and how you are feeling. These questions will take between 5 and 10 minutes to answer. Once this has been done you will be randomly selected to go into one of two groups. One group will be given access to the website training tool, while the other group (the ”waiting list” group) will be told that they will have to wait for 12 weeks before they can have access. Volunteers in both groups will be asked to answer some more questions in 6 weeks time and in 12 weeks time. Again, the questions will take between 5 and 10 minutes to answer. The only contact we will have with you is by email.

[back to top]

What do I have to do if I take part in the research?

We would like you to work through the training programme by logging in to the website. It has 5 modules and we recommend people make time to do one module a week. Each module takes 20-30 minutes to complete. After 6 weeks we will ask you to fill in some more questionnaires, and again after 12 weeks. To fill in the questionnaires will take about 10 minutes. That is all you have to do – the research just involves using a website and filling in online questionnaires. You do not have to travel anywhere or speak to anyone. If you are in the ”waiting list” group then you will need to wait for 12 weeks before you have access to the website.

[back to top]

What is the website that is being tested?

The website is a self-directed training programme which teaches the principles of cognitive behavioural therapy. This means that people can learn useful ways of thinking and behaving to cope with stressful situations.

[back to top]

What are the alternatives for treatment?

This online training programme is not being tested as a treatment. We are seeing whether it can help improve mental wellbeing as an addition to your usual lifestyle. The alternative is to do what you normally do.

[back to top]

What are the side effects of any treatment received when taking part?

This is a training programme on a website. There are no side effects although it will take up some of your time to do the training.

[back to top]

What are the possible disadvantages and risks of taking part?

This is a website training programme. If it doesn't work for you, or you to decide not to continue with it, then it will have used up the time you have spent on the site.

[back to top]

What are the possible benefits of taking part?

If the training programme works then in the short term you may experience an increase in your psychological wellbeing and in the longer term you may have learned more healthy ways of thinking and behaving.

[back to top]

What happens when the research trial stops?

We will send you an email to thank you for taking part. We will also send you a summary of what the research found. We will also send you a link to a website where you can carry on using the training programme free if you want to.

[back to top]

What if there is a problem?

If there is a technical problem with using the site then please contact our email support line on psywellsupport@anu.edu.au. If you have any other kinds of problem then please contact the research team at the University of Warwick (details below).

[back to top]

Will my part in this trial be kept confidential?

Yes. In order to use the website and fill in our questionnaires we need to know your email address but we do not need to know your full name or address. All the information we have on you will be kept confidential and will not be used for any purpose other than this research. When the research has finished we will delete all personal information (i.e. your email address).

[back to top]

What will happen if I don't want to carry on with the study?

You are free to leave the study at any time without giving a reason. If you do decide to leave the study we will send you an email to confirm that you have withdrawn and we will ask for any feedback that you would like to give us (but you do not have to give any).

[back to top]

What will happen to the results of the study?

We will write a report for the Department of Health in England and we will publish the results in scientific journals. No reports will contain any personal information from participants.

[back to top]

Who is organising and funding the research?

The research is being organised by a team at the University of Warwick, and is supported by NHS Choices and the Department of Health.

[back to top]

Who has reviewed the study?

It has been reviewed by the Black Country NHS ethics committee, reference [NUMBER], [CONTACT DETAILS OF REC].

[back to top]

Contact details for further information

If you require further information or if you have any questions please contact the lead researcher, Dr John Powell at the University of Warwick. Email psywell@warwick.ac.uk, telephone 02476574883, or write to: Dr John Powell, Warwick Medical School, University of Warwick, Gibbet Hill Road, Coventry CV4 7AL.

Yes **No**

NHS choices
Your health, your choices

THE UNIVERSITY OF
WARWICK
